# A Case of Feline Eosinophilic Sclerosing Fibroplasia Associated with a Duodenal Mass Responsive to Exclusive Glucocorticoid Therapy

**DOI:** 10.3390/ani15192888

**Published:** 2025-10-02

**Authors:** Mario Pultrone, Dyana Erba, Michela Pugliese

**Affiliations:** 1 Anicura Policlinico Veterinario Roma Sud, 00173 Rome, Italy; erba.dyana@gmail.com; 2 Ospedale Veterinario Lido Roma, 00121 Rome, Italy; 3 Department of Veterinary Sciences, University of Messina, 98168 Messina, Italy; michela.pugliese@unime.it

**Keywords:** FGESF, feline, duodenal mass, eosinophilic inflammation, glucocorticoid therapy

## Abstract

**Simple Summary:**

Feline gastrointestinal eosinophilic sclerosing fibroplasia (FGESF) is a rare inflammatory disease in cats that can mimic cancer due to the presence of firm intestinal masses. In this report, we describe a 6-year-old cat showing chronic vomiting and weight loss, in which FGESF was diagnosed after detecting a duodenal mass during endoscopy. Treatment with oral prednisolone alone led to rapid clinical improvement and complete disappearance of the mass within 30 days. A minor relapse was managed by adjusting the steroid dose. The cat has remained healthy and symptom-free for over a year. This case highlights that FGESF can respond well to medical therapy alone, avoiding the need for surgery.

**Abstract:**

This report describes a case of feline gastrointestinal eosinophilic sclerosing fibroplasia (FGESF) in a 6-year-old spayed female European Shorthair cat presenting with chronic vomiting and weight loss. Endoscopic examination revealed a submucosal duodenal mass, and histopathological evaluation of endoscopic biopsies confirmed a diagnosis of FGESF. The cat was treated exclusively with oral prednisolone (1 mg/kg SID), leading to complete clinical remission within 15 days. Follow-up ultrasonography and endoscopy performed 30 days later confirmed full resolution of the mass, which was replaced by a focal mucosal depression. Histopathology at that site revealed chronic-active lymphoplasmacytic and neutrophilic enteritis with mild fibroplasia. A mild recurrence of duodenal thickening was observed after steroid tapering, which resolved upon dosage adjustment. The patient has remained clinically stable for 15 months with normal imaging and blood parameters.

## 1. Introduction

Feline gastrointestinal eosinophilic sclerosing fibroplasia (FGESF) is a recently recognized inflammatory disease, first described by Craig et al. in 2009 [1]. Histologically, it is defined by thick fibrous trabeculae infiltrated by eosinophils, proliferating fibroblasts, and multinucleated giant cells. Initially considered rare, FGESF is increasingly reported in clinical practice [2]. However, its true prevalence is likely underestimated due to frequent misdiagnosis as neoplasia, especially in mass- forming presentations. Common anatomical sites include the pylorus, proximal duodenum, and ileocecocolic junction, although atypical localizations such as the mesentery, retroperitoneum, and thoracic cavity have also been documented [3,4,5]. The pathogenesis of FGESF remains elusive. More than 50% of cases demonstrate intralesional bacteria on histology or culture, implicating microbial agents as potential triggers [6]. For instance, bacteria were frequently observed in the large retrospective study by Cerna et al., which included 60 cats. Conversely, cases devoid of microorganisms support a sterile or immune-mediated etiology.3 Grau-Roma et al. described fungal phycomycetes within FGESF lesions, suggesting a broader spectrum of potential infectious contributors [7]. The identification of systemic immune activation, as evidenced by gastric lymphoid hyperplasia in multiple cases, including the current one, raises the possibility of hypersensitivity or aberrant immune responses as initiators of eosinophilic inflammation. Notably, Weissman et al. (2021) reported FGESF associated with T-cell/NK-cell lymphoma, raising concern about possible malignant transformation in chronic cases [8]. More recently, Duclos et al. (2023) described a case of intrathoracic FGESF with intralesional Gram-positive bacteria, supporting the hypothesis of bacterial translocation or penetrating injury as pathogenic events [5].

## 2. Case Description

A 6-year-old spayed female European Shorthair cat presented for persistent vomiting and weight loss over three months. Physical examination was unremarkable. Hematological analysis showed marked peripheral eosinophilia (2.9 × 10^9^/L; reference interval 0.17–1.57 × 10^9^/L), while serum biochemistry and urinalysis remained within reference intervals. Abdominal ultrasonography revealed segmental and circumferential thickening of the proximal duodenum, extending approximately 1 cm and forming a subobstructive mass. The lesion was characterized by markedly hypoechoic and thickened submucosa, as well as mild thickening of the muscular layer. The remaining small intestine showed mild, diffuse thickening of the muscularis, with otherwise preserved wall layering and normal motility. The colon was distended with normally shaped intraluminal contents. The stomach displayed diffuse wall thickening, predominantly affecting the muscularis layer. In one area, there was focal loss of normal wall layering, with maximal thickness of 0.45 cm, minimal mucosal discontinuity, and an air-trapping artifact. Mild lymphadenomegaly of the colic lymph nodes was noted, though their internal echotexture remained normal. No free peritoneal effusion was observed. The ultrasonographic pattern was suggestive of FGESF, as described previously [9].

Given the submucosal nature of the lesion, endoscopic biopsy was selected as the initial diagnostic approach rather than surgical exploration because it is less invasive, associated with lower morbidity and faster recovery, and can provide adequate diagnostic yield in mucosal and submucosal diseases when deep samples are obtained. However, this method has the inherent limitation of potentially incomplete sampling of deeper fibrosing components, with the risk of reduced diagnostic accuracy and failure to detect underlying neoplasia. In this case, the decision was supported by the patient’s stable clinical condition, the owners’ preference to avoid surgery, and the availability of an experienced endoscopist able to obtain sufficiently deep and large mucosal and submucosal biopsies.

**Figure 1:**
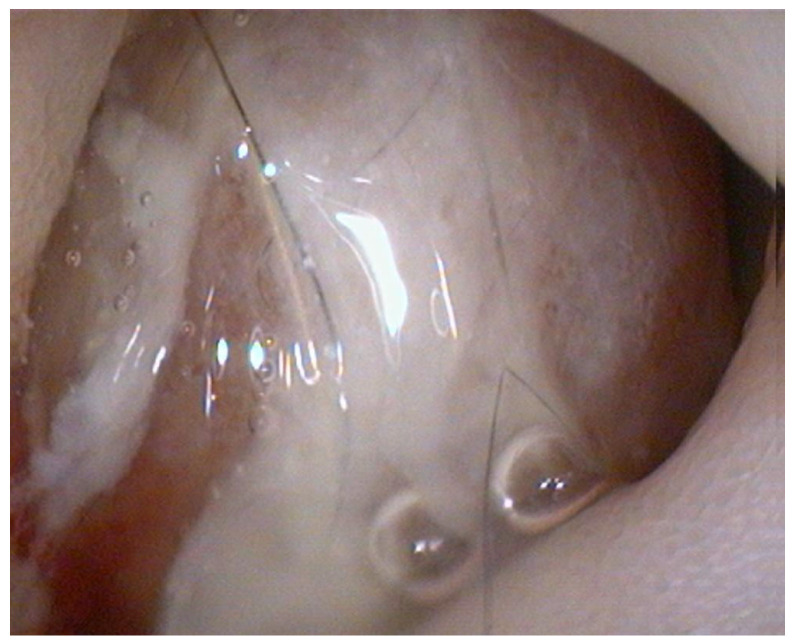
Endoscopic view of the duodenal mass.

Endoscopy, performed using a flexible veterinary gastroduodenoscope (outer diameter 7.9 mm, Karl Storz Silver Scope 60714NKS), confirmed the presence of a submucosal sessile mass partially obstructing the duodenal lumen (Figure 1). It was not possible to fully advance the endoscope beyond the lesion for a complete duodenal evaluation; however, the mucosa visible just distal to the mass displayed shortened and edematous villi. Thanks to the small diameter of the biopsy forceps, it was possible to pass beyond the lesion endoscopically and obtain samples from the duodenal tract immediately distal to the mass. Additional biopsies were taken from the mass itself and from the gastric mucosa, which showed diffuse follicular hyperplasia, using 2.4 mm fenestrated biopsy forceps (Alton AF-D2421BT).

**Figure 2:**
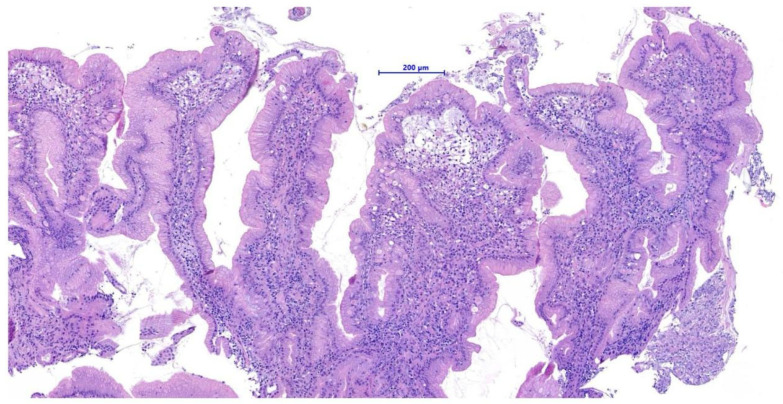
Histological section of endoscopic duodenal biopsy showing dense eosinophilic infiltrates and marked fibroplasia with disrupted mucosal architecture H&E, 200×, scale bar = 200 µm.

Histopathological examination of the gastric biopsies revealed mucosa with deep, hyperplastic crypts and occasional erosive and ulcerated areas replaced by necrotic and collagenous material containing abundant eosinophils, together with a mixed inflammatory infiltrate of neutrophils, small lymphocytes, and scattered eosinophils. Duodenal biopsies, including those obtained distal to the mass, showed irregular, shortened and fused villi with a moderate mixed lymphoplasmacytic infiltrate containing neutrophils and eosinophils in the lamina propria. Histopathological examination of the duodenal mass revealed a sample devoid of recognizable microanatomical architecture, composed of fibrocollagenous tissue interspersed with densely cellular areas (Figure 2). Overall, the findings were consistent with eosinophilic and fibroplastic gastroenteritis compatible with feline gastrointestinal eosinophilic sclerosing fibroplasia (FGESF).

Given the subobstructive behavior of the duodenal lesion and the inherent diagnostic uncertainty associated with mass-forming FGESF, surgical excision was initially proposed as a definitive therapeutic and diagnostic approach.

However, due to economic limitations, the owners declined the surgical option. Consequently, a conservative strategy was adopted, relying exclusively on medical therapy and nutritional support, with the aim of achieving clinical resolution through immunosuppression.

The cat was treated with oral prednisolone (1 mg/kg SID). The diet, already based on a commercial hypoallergenic formulation, was temporarily administered in a liquid consistency by mixing with water, to minimize mechanical aggravation of the obstruction and ensure adequate nutritional intake.

Clinical improvement, with resolution of vomiting and weight stabilization, was noted within 15 days. At 30 days, abdominal ultrasound demonstrated normalization of duodenal thickness. Follow-up endoscopic evaluation was planned from the outset at 30 days after starting prednisolone therapy, which began on the same day as the initial procedure. The aim was to assess the early response to medical treatment and determine whether regression of the duodenal mass could support continuation of a conservative approach, avoiding surgery. This follow-up was performed regardless of the clinical course in order to objectively document lesion reduction, confirm the validity of continued medical management, and obtain additional biopsy samples to exclude alternative diagnoses—particularly lymphoma—and reaffirm the inflammatory nature of the lesion.

**Figure 3:**
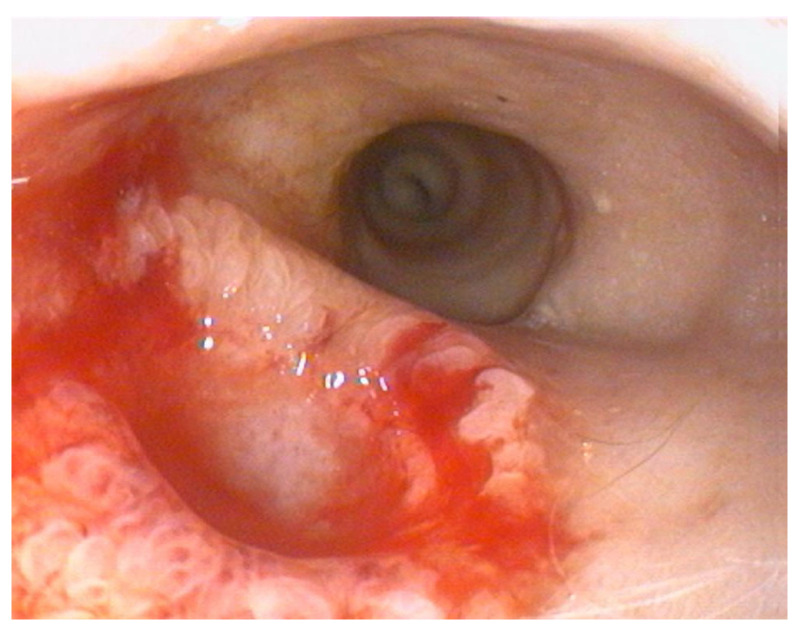
Endoscopic view showing mucosal depression.

The endoscopy demonstrated complete resolution of the duodenal mass, which was replaced by a well- demarcated focal mucosal depression at the site of the previous lesion (Figure 3).

**Figure 4:**
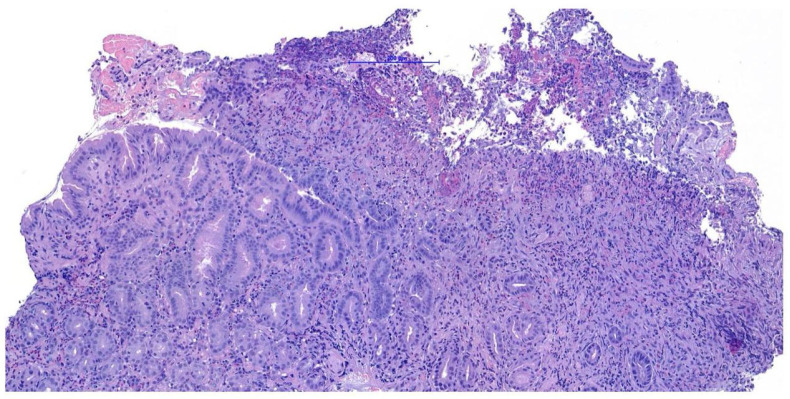
Histological section: moderate chronic-active lymphoplasmacytic and neutrophilic enteritis with fibroplasia H&E, 200×, scale bar = 200 µm.

Endoscopic biopsies collected from this area revealed coalescent villi with thickened and edematous apices, without damage to the surface epithelium. Occasional glandular dilatation was observed, indicating mild epithelial injury. No lymphatic vessel dilation was present. Mucosal fibroplasia was mild. The number of intraepithelial lymphocytes was within normal limits, while the lamina propria showed moderate infiltration of lymphocytes and plasma cells, along with a mild increase in neutrophils. These findings were interpreted as consistent with a moderate chronic- active lymphoplasmacytic and neutrophilic enteritis with mild fibroplasia, suggestive of a post- inflammatory reparative phase, consistent with the resolution of previously diagnosed FGESF (Figure 4).

Histopathological evaluation of both the initial duodenal mass and the follow-up biopsies showed no lymphoid atypia or features consistent with lymphoma.

Prednisolone was tapered to 0.5 mg/kg every 48 h. After 30 days at the lower dose, a mild recurrence of duodenal thickening was noted on ultrasound, although the cat remained asymptomatic. The prednisolone dosage was adjusted to 0.5 mg/kg SID, with subsequent stabilization of ultrasonographic findings. Fifteen months after diagnosis, the patient maintained on a commercial hypoallergenic diet remains in clinical remission. Monthly abdominal ultrasound examinations confirm the absence of duodenal mucosal abnormalities, while hematological and biochemical profiles are monitored every six months, given the lack of clinical signs and to limit costs for the owners. To date, blood tests have shown no eosinophilia or significant biochemical abnormalities.

## 3. Discussion

This case highlights the therapeutic potential of exclusive medical management in feline FGESF, even in the presence of a subobstructive duodenal mass. Historically, the disease has been managed surgically due to its obstructive nature and diagnostic ambiguity, with several studies including those by Linton et al. [2]. and Cerna et al. [6] supporting surgical resection as a definitive or confirmatory option. Similarly, Kambe et al. described a case requiring surgical excision due to the lesion’s mesenteric location and diagnostic complexity [3]. In conclusion, the main ultrasonographic feature of FGESF is a focal, heterogeneous gastrointestinal mass, which commonly shows hyperechoic areas and is most commonly associated with loss of intestinal layering, circumferential thickening, and eccentric growth. These features are similar to gastrointestinal tumors, but the presence of hyperechoic areas within a mass may be strongly suggestive of FGESF rather than neoplasia. Recurrence rates were not different between cases diagnosed with surgical excision and those diagnosed with nonexcisional biopsy. Progression was more frequently described in cats diagnosed by nonexcisional biopsy. Oscillation of ultrasound findings appeared common in this large group of FGESF patients, and correlation with clinical signs seemed inconsistent.

However, our case questions the traditional paradigm of surgical management, showing that endoscopic biopsies can be diagnostically adequate even for mass-forming lesions, and that corticosteroid therapy alone may achieve full remission. This observation is noteworthy, as it suggests that, in selected cases and under appropriate clinical monitoring, mass-forming FGESF lesions may respond to medical therapy alone. Although this is a single-subject report, it supports a possible therapeutic shift toward non-invasive management, provided that diagnostic confidence is high and careful monitoring is maintained. Prospective studies are warranted to identify which cases are amenable to medical therapy alone and to determine predictive markers of response. A major challenge in FGESF is the risk of misdiagnosis, especially when submucosal infiltration is not adequately sampled. Mucosal biopsies may miss deeper fibrosing lesions or mimic neoplastic processes such as metastatic carcinoma or mast cell tumors. Grau-Roma et al. stressed the need for deep sampling when fungal elements are suspected, and Weissman et al. further highlighted diagnostic overlap with lymphoproliferative diseases [7,8]. In their study, three cats had FGESF lesions histologically mimicking lymphoma, and in some cases, true T-cell/NK-cell lymphoma was diagnosed. The eosinophilic inflammation and fibrosis were initially interpreted as reactive, delaying correct identification of the neoplastic component. These observations underscore the need for immunohistochemistry and expert histopathological interpretation when FGESF-like lesions exhibit atypical features or incomplete treatment response. In our case, the absence of lymphoid atypia, the rapid and complete response to glucocorticoids, and the long-term clinical stability argue against a neoplastic process. Nonetheless, the potential for overlap or progression from FGESF to lymphoma, as postulated by Klang et al. [8]. and Cerna et al. [7], mandates long- term vigilance. It is biologically plausible that chronic eosinophilic inflammation may promote oncogenesis through persistent cytokine stimulation (e.g., IL-5, IL-13, eotaxin). The lymphoid aggregates observed in this case may thus reflect early immune dysregulation, further justifying imaging and histologic follow-up even in asymptomatic patients. A limitation of this study is the inability to perform special stains and microbial cultures for bacteria and fungi, as the owners declined further diagnostic costs. However, the complete remission achieved with corticosteroid therapy alone, without antimicrobial treatment, strongly suggests a sterile form of FGESF. This aligns with findings by Cerna et al. [6] where 43% of cases lacked identifiable microorganisms, both histologically and microbiologically. Nonetheless, other reports such as those by Duclos et al. [5] and Grau-Roma et al. [7] have documented the presence of intralesional bacteria or fungi, emphasizing the heterogeneous pathogenesis of FGESF and the clinical relevance of targeted diagnostics when feasible [5,6,7]. The gastric lymphoid hyperplasia observed here, as also reported in previous FGESF cases may reflect systemic immune activation rather than localized infection [2,3,4,5,6,7,8,9,10]. Whether this represents a primary immunological disorder or is secondary to chronic antigen exposure remains unclear. Therapeutically, corticosteroids remain the first-line treatment. In our patient, prednisolone led to rapid clinical and endoscopic remission. However, a mild recurrence upon tapering indicates that FGESF may require prolonged immunosuppression. Cerna et al. reported similar relapses reinforcing the importance of individualized tapering protocols [6]. Other immunosuppressants (e.g., ciclosporin, chlorambucil) have been proposed but remain second line options in the absence of standardized guidelines. Long term therapy must be balanced with careful monitoring to mitigate adverse effects such as diabetes mellitus and secondary infections. Overall, this case supports a broader understanding of FGESF as a spectrum disorder, ranging from sterile mucosal lesions responsive to medical therapy to complex, potentially neoplastic forms requiring surgical or oncological intervention. Increased awareness of this variability is essential for improving diagnostic accuracy and tailoring treatment appropriately, provided that adequate diagnostic sampling and long-term follow-up are feasible.

## 4. Conclusions

FGESF should be considered in cats presenting with gastrointestinal masses and peripheral eosinophilia, especially when ultrasonography reveals segmental thickening with preserved layering. This case demonstrates that even subobstructive mass lesions can be accurately diagnosed through endoscopic biopsy and may regress completely with exclusive glucocorticoid therapy. Although conclusions are necessarily limited by the single-subject design, this report suggests that not all cases of mass-forming FGESF require surgical management. Further investigations are warranted to determine the proportion of such cases amenable to medical therapy alone and to define clinical or imaging predictors of outcome. Adequate biopsy technique, awareness of histological patterns, and individualized immunosuppressive protocols are key to successful management. Given possible associations with lymphoma, vigilant long-term monitoring remains essential.

## Data Availability

Data are contained within the article.

## Abbreviations

The following abbreviations are used in this manuscript:

FGESF	Gastrointestinal Eosinophilic Sclerosing Fibroplasia
